# Uric acid and ammonia play synergistic roles in the loss of blood-brain barrier integrity in the context of hepatic encephalopathy

**DOI:** 10.1186/s12987-025-00746-6

**Published:** 2025-12-13

**Authors:** Sydnée L’Ecuyer, Victoria Yip, Maximilian Steger, Farzaneh Tamnanloo, Mariana Oliveira, Mélanie Tremblay, Christopher F. Rose, Emmanuel Charbonney

**Affiliations:** 1 Departement of Pharmacology and Physiology, Université de Montréal, Montreal, Canada; 2 Hepato-Neuro Lab, Centre de Recherche du Centre Hospitalier de l’Université de Montréal, Montreal, Canada; 3 Departement of Neuroscience, Université de Montréal, Montreal, Canada; 4 Departement of Medicine, Université de Montréal, Montreal, Canada; 5 Intensive Care Unit, Centre Hospitalier de l’Université de Montéal, Montreal, Canada

**Keywords:** Blood-brain barrier, Hepatic encephalopathy, Hyperammonemia, Hyperuricemia, Uric acid, Ammonia

## Abstract

**Background:**

The blood-brain barrier (BBB) maintains brain homeostasis by regulating molecular entry. Its endothelial cells are joined by tight junction proteins and express few apical transporters. Hyperuricemia, known to impair tight junctions and endothelial function, may similarly affect the BBB. While ammonia freely diffuses across the BBB, its impact on endothelial integrity is unclear. This study investigates the combined effects of uric acid (UA) and ammonia on BBB function.

**Methods:**

The BBB integrity in bile-duct ligated rats with hyperuricemia was investigated by evaluating permeability to UA, endothelial cell apoptosis and expression of tight junction proteins in the cerebral cortex. To mimic the conditions described in BDL rat, cultured primary rat brain microvascular endothelial cells (RBMEC), cells were exposed to UA (300 µmol/L) and ammonia (200 or 800 µmol/L) for 20 h prior to assessing the endothelial layer resistance, cell viability and expression of tight junction proteins.

**Results:**

Both BDL rats and RBMEC cells exposed to UA and ammonia present with a significant loss of BBB integrity shown by an increased permeability to UA in rats and a decrease endothelial layer resistance in cells. Additionally, both in rats and RBMEC exposure to UA increases endothelial cell apoptosis and exposure to ammonia decreases the expression and integrity of tight junction proteins.

**Conclusions:**

The effects of uric acid on endothelial cell viability and of ammonia on tight junction integrity leads to a combined effect of both these molecules on the integrity of the BBB.

## Introduction

The blood-brain barrier (BBB) is a crucial structure responsible for maintaining brain homeostasis by tightly regulating the exchange of nutrients, toxins, and pathogens between the blood and the brain [1]. The primary structural component of the BBB is the polarized layer of highly specialized endothelial cells [1–3]. To ensure proper function, these endothelial cells contain many mitochondria and are securely connected by tight junction (TJ) proteins [1]. They also feature distinct transporters at the apical and basolateral membranes, enabling precise regulation of molecular and cellular transport in and out of the brain [4]. The loss of integrity of these TJ protein complexes, particularly of the ZO-1 protein could lead to a significant increase in paracellular permeability [5]. The endothelial layer of the BBB is supported by astrocytes and pericytes, acting as intermediaries for communication between endothelial cells and neurons [1, 6].

Hepatic encephalopathy (HE) is a neurological disorder developing as a consequence of liver cirrhosis. In the context of HE, particularly in animal models of HE induced by bile-duct ligation (BDL), studies of the BBB integrity yield varied results, with some studies reporting alterations, while others do not [7]. These discrepancies may stem from the fact that most HE studies primarily focus on ammonia and its distinctive interaction with the BBB as well as the big variability of plasma ammonia in BDL rats ranging from 200 to 800 umol/l [8]. In its gaseous form, it can diffuse freely through cellular membranes. Whereas, in its ionic form, it can be transported through potassium channels, the Na^+^/K^+^ ATPase or the KCl transporter due to the similar ionic radius and charge between K^+^ and NH_4_^+^ ions [9]. This dual transport mechanism facilitates ammonia entry into the brain, where it is then metabolized into glutamine by astrocytes [10]. At high levels, circulating ammonia may increase oxidative stress in the endothelial layer of the BBB, leading to mitochondrial dysfunction [11], potentially affecting the integrity of the barrier. Additionally, even though ammonia is not known to affect the endothelial cells themselves, hyperammonemia can affect astrocytes, an integral part of the BBB structure, causing BBB dysfunction and breakdown [12].

Recent studies into the incidence of chronic liver disease and its aetiologies have shown an increase in metabolic-associated steatotic liver disease [13]. These patients also frequently present with metabolic syndrome (MetS). One of the cornerstones of MetS pathophysiology is increased circulating uric acid (UA) values qualified as hyperuricemia [14]. This increased circulation of UA could be problematic for BBB integrity because hyperuricemia also causes endothelial dysfunction [15, 16].

To simulate the role of hyperuricemia in HE pathophysiology, we previously administered a diet containing 3% UA in rats with a bile-duct ligation. In these rats, we showed that hyperuricemia, with values of circulating UA around 300 umol/l contributed to cognitive impairment and neuronal cell loss (*L’Ecuyer et al.*. 2025. under review). Knowing that UA is known to contribute to endothelial dysfunction by scavenging nitric oxide (NO) and decreasing the activity of the nitric oxide synthetase (eNOS) in human umbilical vein endothelial cells (HUVEC) [17, 18], the present study focuses on the potential effects of hyperuricemia on BBB integrity in the context of HE. Given the highly polarized nature of the BBB endothelium, characterized by the presence of tight junctions between cells inducing polarization, it is reasonable to hypothesize that the BBB may respond similarly to epithelia. Hyperuricemia affects the integrity of TJ proteins in both the intestinal [19] and renal epithelium [20] affecting the integrity of the protective layer. UA can also act dually as an antioxidant and a pro-oxidant molecule depending on the setting [21]. One of the primary factors that triggers UA to act as a pro-oxidant is the presence of a pro-oxidant setting [21] that is known to occur in individuals with HE [22].

Given the overlapping mechanisms of oxidative stress and barrier disruption, it is reasonable to consider that ammonia and uric acid may have combined effects on the integrity of the BBB in individuals with HE. Indeed, by inducing oxidative stress, hyperammonemia may trigger UA to induce further damage to the endothelial layer. This study focuses on investigating the joint effects of hyperammonemia and hyperuricemia on blood-brain barrier integrity in both a rat model of HE induced by BDL and cultured primary rat brain microvascular endothelial cells to allow for an isolated investigation of the effects of UA and ammonia alone as well as the combined effects of UA and ammonia.

## Methods

### Experimental design

#### Animal experiments

Male Sprague-Dawley rats (*n* = 32, 190–225 g; Charles River) were fed *ad libitum* a diet containing 3% uric acid (HUA; Envigo) or a regular diet (RD; Envigo). Three days after arrival the rats underwent SHAM or BDL surgery as previously described by our team [8]. The rats were then assigned to one of four experimental groups: (1) SHAM with RD (*n* = 6; SR), (2) SHAM with HUA (*n* = 6; SUA), (3) BDL with RD (*n* = 6; BR), (4) BDL with HUA (*n* = 6; BUA). All experiments were approved by the Institutional Animal Care and Use Committee at the CRCHUM (4I015049CR). One hour before surgery pain management was administered with a dose of 5 mg/kg of carprofen and 0.5 mg/kg of buprenorphine SC and 60 mg/kg of gabapentin orally. At time of surgery, anesthesia was induced with 4% isoflurane and maintained with 2-2.5% of isoflurane in 1 ml/min oxygen. Buprenorphine and gabapentin were readministered at the end of the surgery day. Animals were then closely monitored for 5 days post-surgery with administration of 5 mg/kg carprofen daily for the first two days, followed by daily monitoring of weight and hydration levels with saline administered SC as required. To ensure continued animal welfare, animal weight and SC vitamin K administration were completed weekly. At day 33 post-surgery, animals were sacrificed by isoflurane overdose and cardiac puncture, total blood was collected was separated by centrifugation. Plasma was then aliquoted, and brains were dissected to extract cortex, before being snap frozen in methylbutane and kept at -80 °C until analysis. A separate set of 2 animals per group were perfused with 10% formalin before tissue collection. The brains from these rats were cryopreserved in a 3% sucrose solution, embedded in optimal cutting temperature compound (OCT), and stored at -80 °C for immunofluorescence analysis on coronal brain cryosections. All model characteristics concerning the concentration of plasma markers, liver disease severity, cognitive impairment and neuronal damage are presented in *L’Ecuyer et al.* (Under Review).

#### Cell experiments

Primary rat brain microvascular endothelial cells isolated from the cortex of male rats (RBMEC; R1078; Cell Biologics) were used from the 6th to 11th passage. After thawing, the cells were washed with PBS (311 − 013 CL; Wisent Multicell) at 37 °C and suspended in fresh rat brain endothelial cell media (M1266; Cell Biologics). The cells were cultivated at 37 °C in 5% CO_2_ in a humidified incubator. After being expanded in T75 flasks, cells were seeded in their respective container for treatment described in Table 1. UA is solubilized in 0.1 N NaOH and ammonium chloride is solubilized in 1x PBS. To ensure proper validity of the control, Vehicle treated cells were exposed to the highest concentration of 0.1 N NaOH and 1x PBS to eliminate all potential effects associated with the solvents.

**Table 1:** Treatment conditions for rat brain microvascular endothelial cells

	Individual				Combined	
Condition	*Vehicle*	*UA 300*	*NH*_*4*_ *200*	*NH*_*4*_ *800*	*UA 300 + NH4 200*	*UA 300 + NH4 800*
Uric acid (µmol/L)	0	300	0	0	300	300
Ammonia (µmol/L)	0	0	200	800	200	800

### Uric acid concentration measurements

Plasmatic and cortical UA were assessed using the COBAS c111 analyzer (Roche Diagnostics).

Plasma samples isolated at sacrifice were loaded as is to be evaluated for UA concentrations. A lysate of cortexes was prepared by sonication in RIPA buffer (Tris HCl pH 7.4: 50 mM; NaCl: 150 mM; EDTA: 1 mM; SDS: 0.1%; Triton X-100: 1%; Proteinase inhibitor cocktail (P8340; Sigma; 1:500)) and protein concentrations were assessed using the Lowry method. A volume equivalent to 200 ug of protein for each sample were loaded in the COBAS c111 for UA concentration assessment.

Cortical samples were not perfused prior to dissection but a ratio to control ensures that the UA concentration in residual blood is considered for all animals.

### Transendothelial resistance evaluation using the ECIS-Z-θ system

Transendothelial resistance (TEER) was monitored using the ECIS-Z-θ system (Applied Biophysics). Two 8W10E + arrays (Applied Biophysics) are coated with collagen Type IV (C6745, Sigma-Aldrich). The cells are seeded at a density of 75.000 cells/cm^2^. A confluent barrier formed after 24 h post seeding. Upon barrier formation, culturing media was replaced with treatment (Table 1) in DMEM. The resistance is then monitored for 20 h and the areas under the curve are analyzed using the GraphPad Prism 10.0.2 Software.

### In vitro fluorescein sodium salt permeability assay

To assess the permeability, fluorescein sodium salt (NaF) is used. 50.000 cells/cm^2^ are seeded on 24 well 0.4 mm pore size Transwell inserts (CLS3470; Millipore Sigma) and allowed to reach confluency. After the cells are exposed for 20 h to the previously described treatments (Table 1), the media in the apical compartment is replaced with 100 µg/mL of NaF in DMEM. Samples are taken every 15 min both from the apical and basolateral sides. The intensity of the fluorescence is measured at an excitation of 485 nm and emission of 525 nm using a fluorometric plate reader.

### Nitric oxide colorimetric assay

For nitric oxide analysis in both cell culture media and rat plasma the samples were first deproteinated and the nitric oxide colorimetric detection kit (EIANOC; Invitrogen) was used following the manufacturer’s protocol.

### Real-Time Glo cell viability assay

Real-Time Glo cell viability assay (G9711; Promega) reagent was added to cells seeded at a 50.000 cells/cm^2^ density in a 96 well plate at the same time as the treatment (Table 1) and the assay was performed according to the manufacturer’s protocol.

### Alamar blue cell viability assay

RBMECs are seeded at a 50.000 cells/cm^2^ density in 96-well plates and incubated at 37 °C with the treatment in triplicates for 20 h. The treatment is replaced with 440 µmol/l Alamar Blue in DMEM and incubated for 4 h at 37 °C. Fluorescence is measured at excitation of 560 nm and emission of 590 nm in a fluorometric plate reader.

### MitoSOX analysis

MitoSOX Red (M36007; Invitrogen) staining is performed on cells seeded at a 50.000 cells/cm^2^ density in a 96 well plate and on a microscope slide with the 12 well FlexiPerm inserts (94.6011.436; Sarstedt) following the manufacturer’s protocol. Fluorescence is evaluated in a fluorometric plate reader at an excitation of 396 nm and an emission of 610 nm. After staining, cells on the FlexiPerm slide were fixed in formalin for 10 min and incubated with DAPI (4′,6-diamidino-2-phenylindole) for 10 min before being imaged using the Zeiss AxioImager M2 microscope.

### Caspases activity assay

The activity of caspases-3, 8 and 9 are assessed by enzyme assay following the procedure described in *L’Ecuyer et al. 2020* [23] using 25 ug of protein by sample.

### Protein lysate preparation

One hemisphere of the cortex and 1 well of a 6 well plate exposed to treatment conditions described in Table 1 were collected and placed in RIPA Buffer (Tris HCl pH 7.4: 50 mM; NaCl: 150 mM; EDTA: 1 mM; SDS: 0.1%; Triton X-100: 1%; Proteinase inhibitor cocktail (P8340; Sigma; 1:500). The samples were then sonicated, incubated on ice for 30 min and centrifuged at 13 000 x g for 15 min at 4 °C. The supernatant was transferred in a new tube and protein concentration is assessed using the Lowry method. The samples were then aliquoted in 20 uL per tube kept at -80 °C before being thawed for use in ELISA and Western Blot experiments.

### CD31/PECAM-1 ELISA

PECAM-1 ELISA is performed on 200 ug of protein loaded in a PECAM-1 ELISA kit (EKU06712; Biomatik Corporation) following the manufacturer’s protocol.

### Western blot analysis

A total of 50 ug of protein is loaded in a 7% SDS-PAGE gel containing 5% trichloroethanol for total protein detection and migrated for 1 h at 150 V. The trichloroethanol was then activated with the ChemiDoc MP Imager (Bio-Rad) to allow detection of total protein staining. Transfer is performed using the Fisherbrand Semidry Blotting Apparatus at 5 V for 1 h to a nitrocellulose membrane 0.45 mm pore size (1620115; Bio-Rad) using transfer buffer (Tris: 25 mM; Glycine: 19.2 mM; Methanol: 20%).

After transfer, the total protein images are taken using the “Stain-free blot” setting on the Chemidoc MP Imager. The membranes are then incubated in blocking buffer (Tween20: 0.05% and Milk powder: 5% in TBS (Tris pH 7.5: 1 mM; NaCl: 10 mM)) for 30 min and washed 3 times in washing buffer (Tween20: 0.05% in TBS 1X). Membranes are incubated with a rabbit polyclonal antibody raised against ZO-1 (402200; Invitrogen; 1:750) for 2 h at room temperature under agitation. The washing step is repeated, and the membranes are incubated with the peroxidase AffiniPure goat anti-rabbit IgG (1:10 000; 111-035-003, Jackson ImmunoResearch Laboratories Inc) for 1 h at room temperature under agitation. One last washing step is performed, and the membranes were then exposed to Clarity Western ECL substrate (1705061, Bio-Rad) before chemiluminescence imaging at the Chemidoc MP Imager.

The ratio of band density to total protein density is analyzed using the Image Lab Software 6 (Bio-Rad) and reported as % to the average of the SR group.

### Immunohistochemistry

Fifteen µm thickness of cortical tissue are cut using Leica CM3050 S cryostat and mounted on Fisherbrand Superfrost Plus Microscope Slides (22-037-246, FisherScientific). RBMEC cells are seeded on microslides (3800240; Leica Biosystems) covered with a 12 well FlexiPerm inserts.

To detect endothelial cell apoptosis, cortex slices wells are immunostained, following the protocol described in *L’Ecuyer et al.*(231), for CD31 (1:250; AF3628; R&D Systems) detected with donkey anti-goat IgG secondary antibody AlexaFluor 647 (1:1000; A-21447; Invitrogen) or for Erg (1:200; MA5-26245; Invitrogen) detected with goat anti-mouse IgG cross-adsorbed secondary antibody AlexaFluor 568 (1:1000; A-11004, Invitrogen). Both endothelial cell markers are co-stained with cleaved caspase-3 (1:250; 9661 S, Cell Signaling Technologies) detected using goat anti-rabbit IgG cross-adsorbed secondary antibody AlexaFluor 488 (1:1000; A-11008, Invitrogen). RBMEC cells slides are only stained using the cleaved-caspase-3 antibody.

To detect changes in barrier integrity in RBMEC cells, immunostaining for ZO-1 (1:200) detected with goat anti-rabbit IgG cross-adsorbed secondary antibody AlexaFluor 488 is also performed.

All slides were counterstained with DAPI to detect the cell nuclei. Ten pictures per condition were taken using the Zeiss AxioImager M2 microscope at a magnification of 40X.

### Statistical analysis

All statistical analysis were performed using the IBM SPSS Statistics 29 software and all graphs and figures are made using BioRender. Data is presented as mean ± standard deviation. Before statistical analysis, all data is assessed for normal distribution using the Shapiro-Wilk test and, if necessary, data is normalized by applying a log to all values. For experiments in rats, data were analyzed using a Two-Way ANOVA followed by MANOVA partitioning to detect interactions between the diet and surgery factors. For cell experiments, data was analyzed using a One-Way ANOVA to compare the influence of individual and combined treatments of UA and ammonia.

## Results

### Impact of diet-induced hyperuricemia on brain uric acid levels in BDL rats

**Fig. 1:**
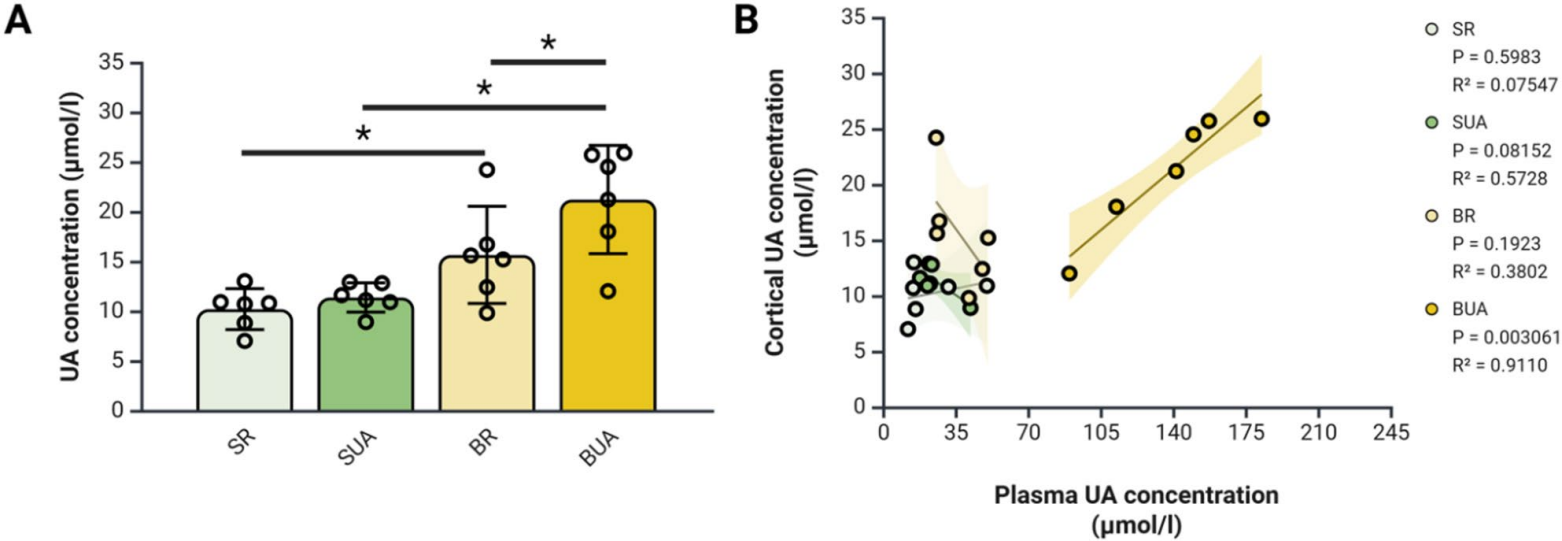
Hyperuricemia in bile-duct ligated rats leads to a significant increase cortical uric acid concentration. (**A**) Cortical uric acid concentration measured by the COBAS c111. (**B**) Group-dependant correlation between plasma and cortical uric acid concentration. *N* = 6 per group; *p < 0.05 with two-way ANOVA; Pearson’s two-tailed correlation; BR: BDL + regular diet; BUA: BDL + high uric acid diet; SR: SHAM + regular diet; SUA: SHAM + high uric acid diet; UA: uric acid. Created with BioRender

Knowing that, in healthy individuals, UA is unable to cross the BBB, as a first sign of BBB breakdown we investigated cortical UA concentrations in all our rats. Cortical UA values are significantly increased in BR animals compared to SR animals (Fig. 1A). Furthermore, BUA animals show a further significant increase when compared to SUA and BR animals (Fig. 1A). These results are the first indication that BBB integrity may be impaired in BR and BUA rats with a further impairment in BUA rats.

However, a correlation between plasma and cortical UA values is present only in BUA rats suggesting free diffusion of UA between the blood and the brain in these animals (Fig. 1B). To validate the joint effects of ammonia, the main pathogenic factor in BDL rats, and uric acid, we then investigated the barrier function of RBMEC exposed to both these molecules.

### The effect of uric acid and ammonia on endothelial barrier resistance and permeability in rat brain microvascular endothelial cells

**Fig. 2:**
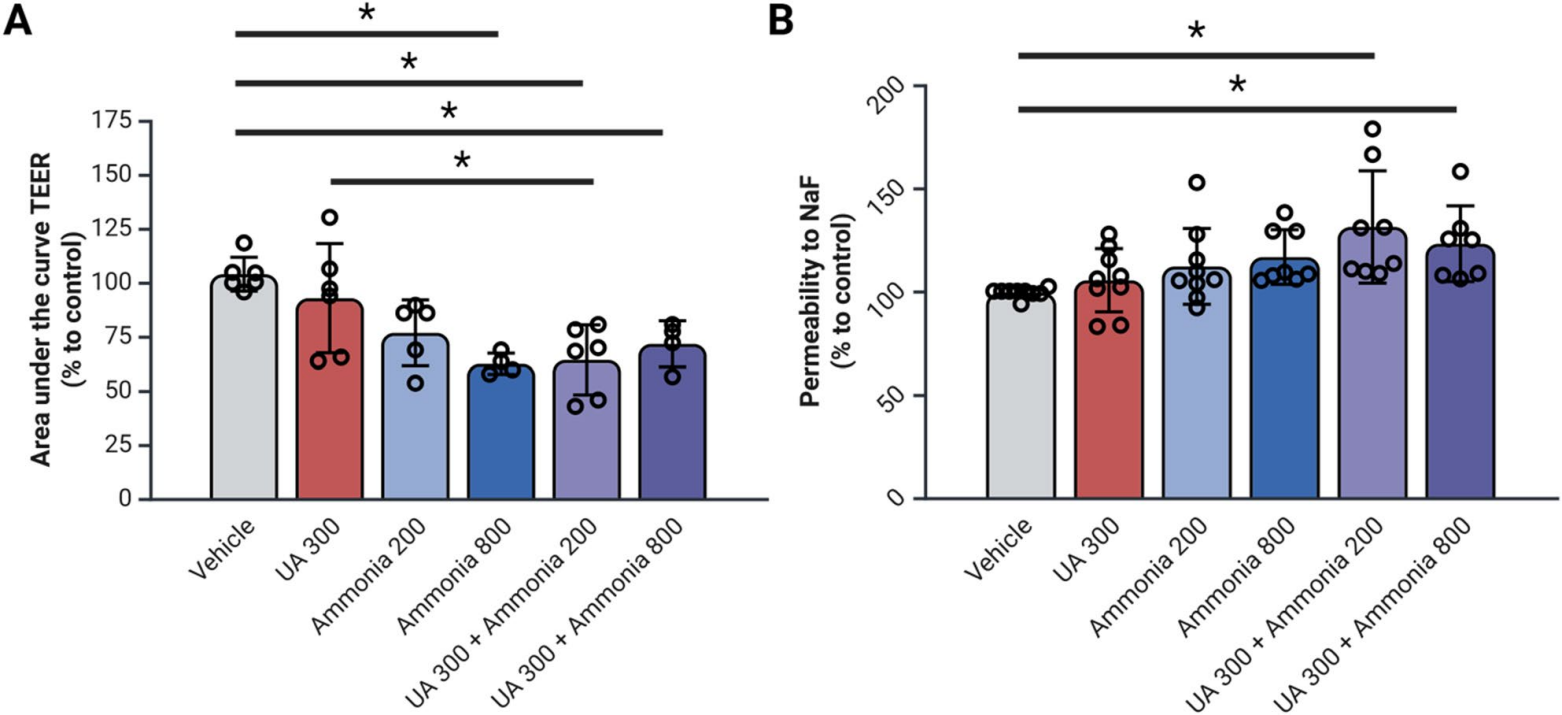
Combined hyperuricemia and hyperammonemia leads to decreased endothelial layer resistance and increased endothelial layer permeability. (**A**) Transendothelial resistance measured by the ECIS-Z-θ of monolayer RBMEC cells cultivated on electrodes and exposed to uric acid and ammonia for 20 h. (**B**) Fluorescein sodium salt permeability assay on monolayer RBMEC cells seeded on Transwell permeable filters and exposed to uric acid and ammonia for 20 h. *N* = 6–8 per condition; *p < 0.05 by One-Way ANOVA followed by Tukey’s post-hoc; NaF: fluorescein sodium salt, NH_4_: ammonia, RBMEC: rat brain microvascular endothelial cells, TEER: transendothelial resistance, UA: uric acid. Created with BioRender

As a parallel to the increased permeability data detected in BUA rats in Fig. 1, we then evaluated the integrity of the endothelial barrier in response to UA, ammonia, and combined treatments. We first looked at the TEER of our endothelial barrier measured by the ECIS-Z-θ system over 20 h with a measure of the area under the curve. The ammonia 800, the UA 300 + ammonia 200 and the UA 300 + ammonia 800 show a significant decrease in AUC when compared to vehicle treated cells (Fig. 2A). Additionally, the UA 300 + ammonia 200 treated cells show significantly lower TEER AUC then the UA 300 treated cells (Fig. 2A).

To complement the results obtained from the TEER measurements, we also measured the permeability to NaF of an RBMEC monolayer seeded on 24 well permeable Transwell inserts. Both the UA 300 + NH_4_ 200 and the UA 300 + NH_4_ 800 treated cells show a significant increase in NaF permeability compared to vehicle (Fig. 2B) supporting the conclusion that UA and ammonia have a joint effect on BBB integrity.

### Impact of uric acid and ammonia on endothelial dysfunction in BDL rats and cultured RBMECs

**Fig. 3:**
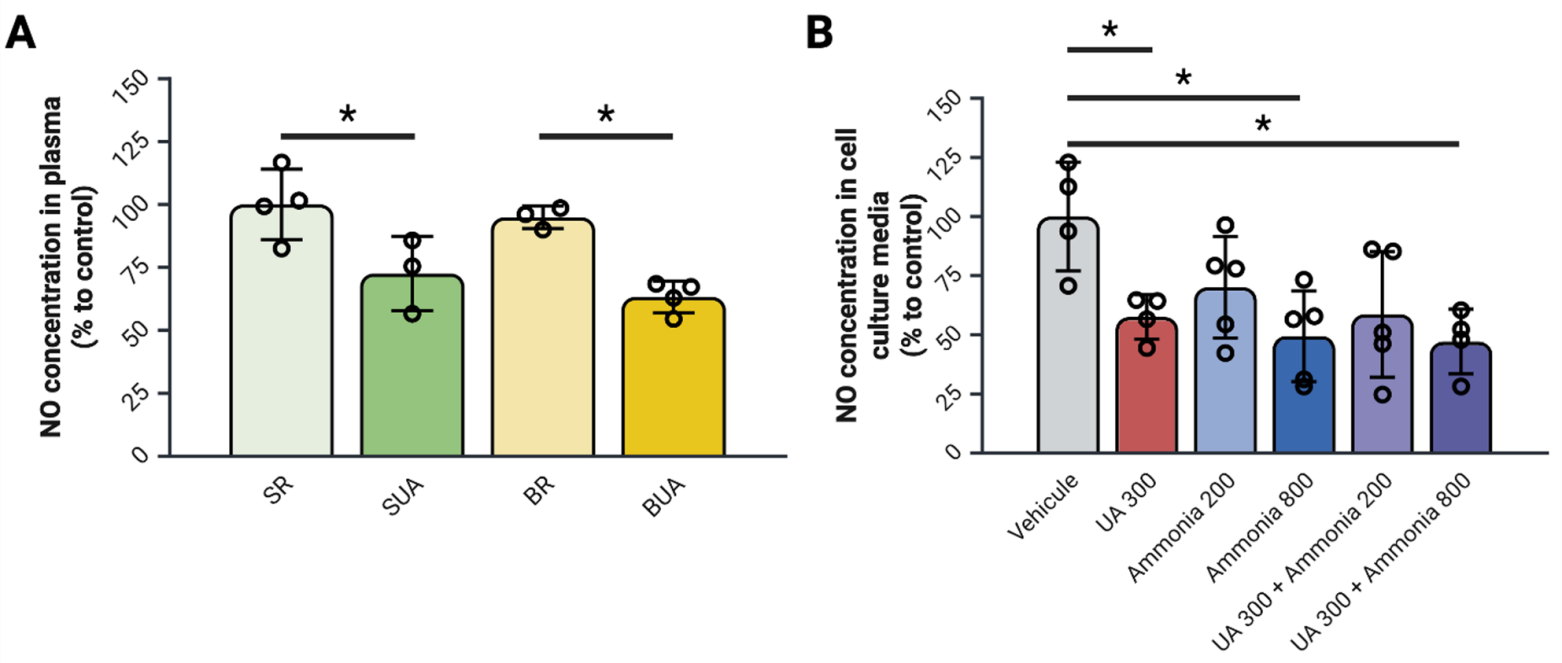
Hyperuricemia in bile-duct ligated rats and endothelial cells exposed to ammonia leads to endothelial dysfunction. (**A**) Nitric oxide concentrations in the plasma of bile-duct ligated rats. (**B**) Nitric oxide concentrations in the cell culture media of RBMEC cells cultivated in a 6 well plate exposed to uric acid and ammonia for 20 h. *N* = 4–6 per condition; *p < 0.05 Two-Way ANOVA for rats; *p < 0.05 One-Way ANOVA with Tukey’s post-hoc for RBMEC cells; BR: BDL + regular diet, BUA: BDL + high uric acid diet, NH_4_: ammonia, NO: Nitric oxide, SR: SHAM + regular diet, SUA: SHAM + high uric acid diet, RBMEC: rat brain microvascular endothelial cells, UA: uric acid. Created with BioRender

Knowing hyperuricemia is associated with endothelial dysfunction, we then looked at circulating and media NO concentrations. In both BDL and SHAM rats, exposure to UA diet leads to a significant decrease in plasma NO concentration compared to their counterparts (Fig. 3A). In RBMECs, exposure to 300 of UA, 800 of ammonia alone and to a combination of UA 300 + Ammonia 800 leads to a significant decrease of NO in the cell culture media when compared to vehicle treated cells (Fig. 3B). These results support that hyperuricemia alone could contribute to dysfunction of the BBB endothelium both in rats and in cells exposed to UA.

### Endothelial cell loss in response to uric acid and ammonia in the context of hepatic encephalopathy

#### Increased cortical endothelial cell loss is detected in bile-duct ligated rats with hyperuricemia

**Fig. 4:**
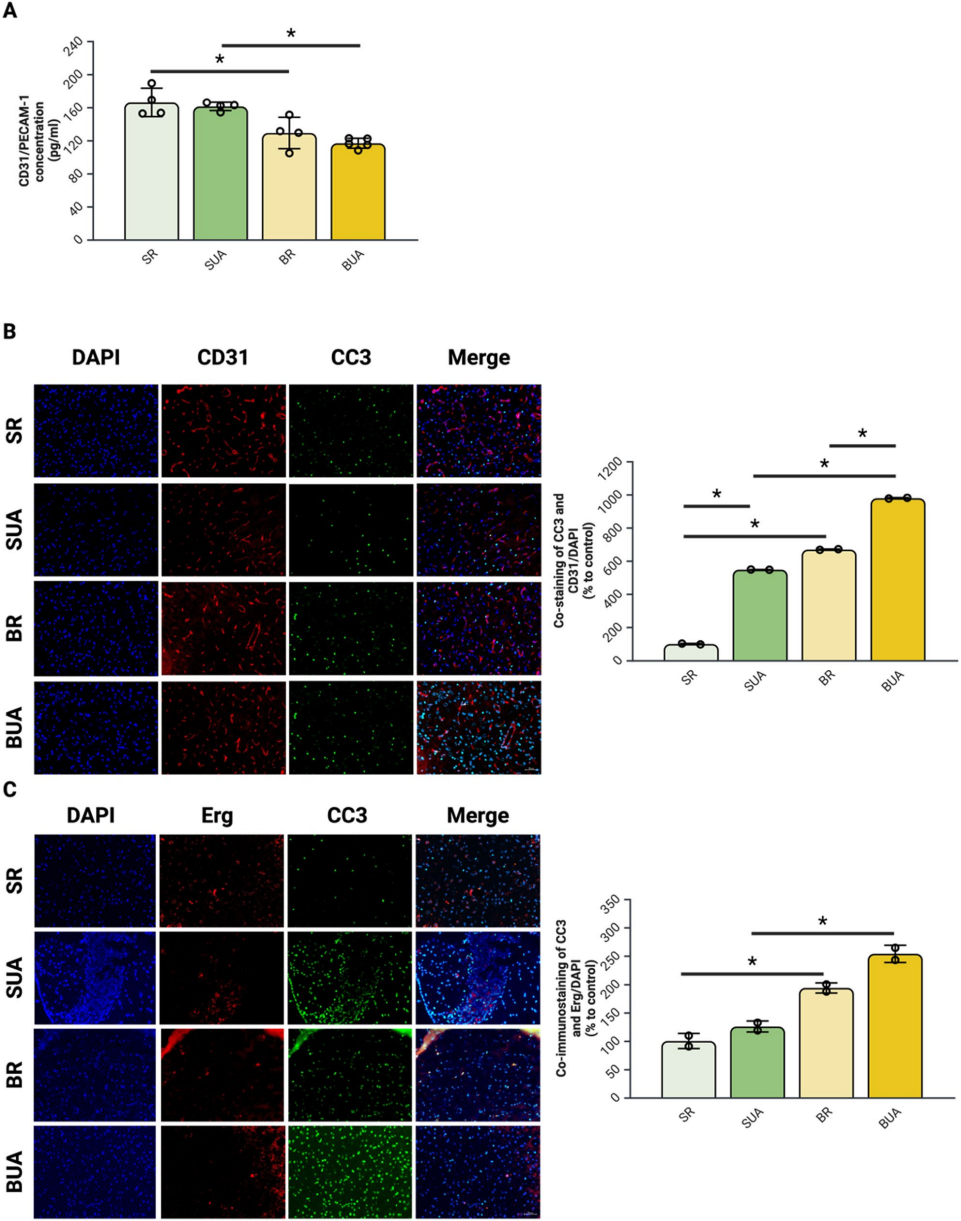
Hyperuricemia in bile-duct ligated rats leads to an increase in cortical endothelial cell loss. (**A**) CD31/PECAM-1 ELISA in the cortex of rats. (**B**) Co-immunostaining of CD31/PECAM-1 and cleaved caspase-3 in the cortex of rats. (**C**) Co-immunostaining of Erg and cleaved caspase-3 in the cortex of rats. *N* = 2–4 per group; *p < 0.05 Two-Way ANOVA; Scale 50 um; BR: BDL + regular diet, BUA: BDL + high uric acid diet, CC3: cleaved caspase-3, CD31: cluster of differentiation 31, Erg: ETS-related gene, SR: SHAM + regular diet; SUA: SHAM + high uric acid diet; UA: uric acid. Created with BioRender

Based on the detected altered permeability in BUA rats, we then investigated whether this alteration was associated with increased endothelial cell loss. When quantifying CD31/PECAM-1 expression by ELISA, we detected a significant decrease for both BR and BUA animals when compared to their SHAM counterparts (Fig. 4A).

We then did co-immunostaining of endothelial cell markers (CD31/PECAM-1 and Erg) and a marker for apoptosis (CC3). Co-immunostaining between CD31/PECAM-1 and CC3 show a significant increase in both BR and BH groups compared to SR animals. Furthermore, BH animals show significant co-staining compared to BR animals (Fig. 4B). Co-immunostaining between Erg and CC3 show a significant increase in both BR and BH group when compared to SR (Fig. 4C). No other differences were detected between groups for this staining.

#### Co-exposure to uric acid and ammonia leads to a significant decrease in endothelial cell viability

**Fig. 5:**
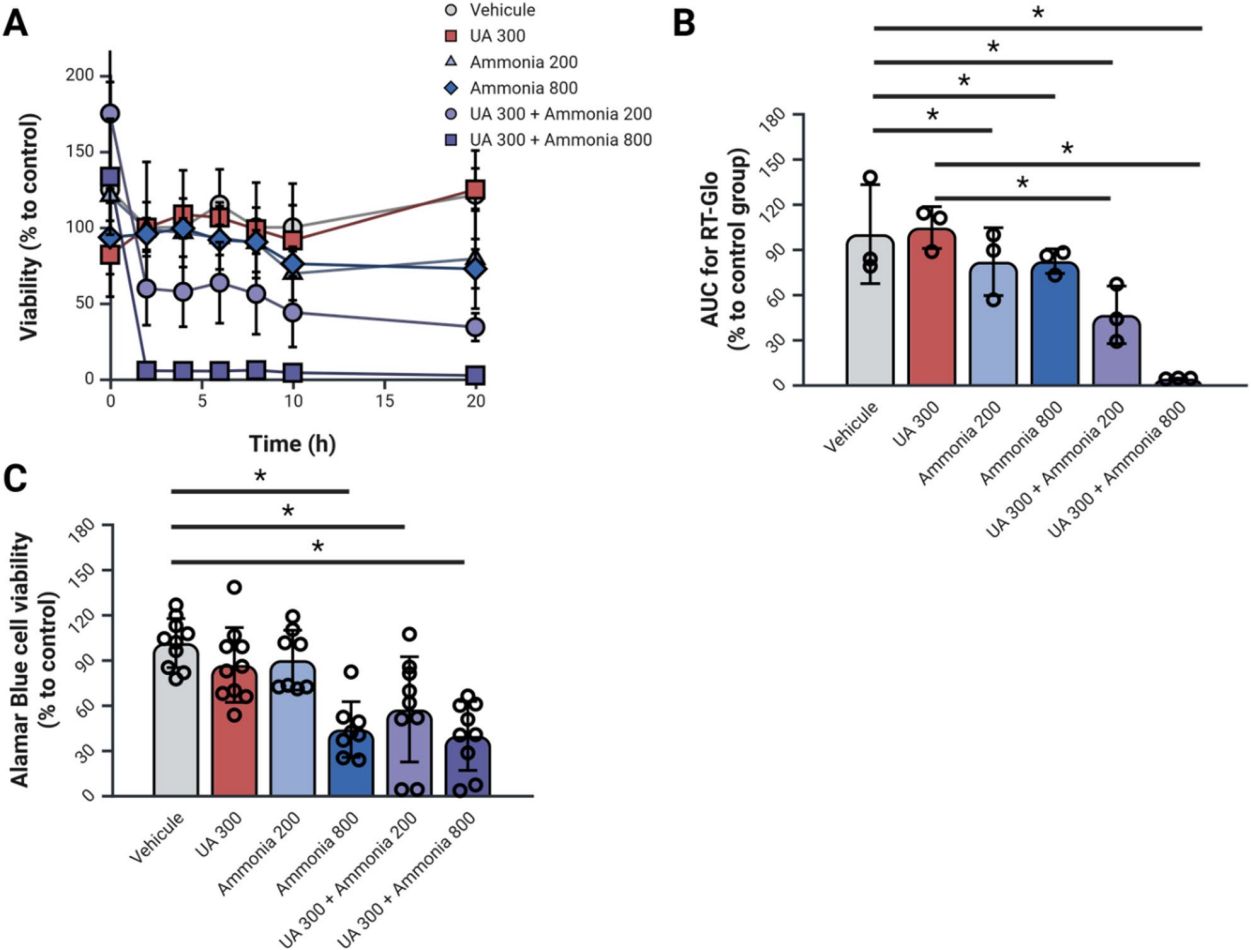
Combined hyperuricemia and hyperammonemia leads to decreased cell viability. (**A**) Real-Time Glo cell viability assay average curve per triplicate according to time with RBMEC cells cultivated on a 96 well plate and exposed to uric acid and ammonia for 20 h. (**B**) Area under the curve of Real-Time Glo cell viability assay curves presented in panel A. (**C**) Alamar Blue assay for RBMEC cells exposed to uric acid and ammonia for 20 h. *N* = 3–8 per condition; *p < 0.05 One-Way ANOVA with Tukey’s post-hoc; NH_4_: ammonia, RBMEC: rat brain microvascular endothelial cells, RT-Glo: Real-Time Glo cell viability assay, UA: uric acid. Created with BioRender

As a parallel to the increased apoptosis detected in BUA rats, cell viability tests were performed on RBMEC cells exposed to UA and ammonia. To assess RBMEC cell viability in response to UA and ammonia treatments according to time we ran a Real-Time Glo cell viability assay (Fig. 5A-B). With this assay, we detected that all ammonia individual treatments as well as combined treatments of UA and ammonia showed a significant decrease in cell viability when compared to vehicle. Additionally, both UA + ammonia combined conditions showed a significant decrease in cell viability when compared to the UA 300 treated cells.

We also performed the Alamar Blue assay at 20 h after the beginning of the treatment. The cells treated with ammonia 800 and the combined treatments of UA and ammonia show a significant decrease in cell viability with the Alamar Blue assay compared to the vehicle treated cells (Fig. 5C).

#### Co-exposure to uric acid and ammonia leads to a significant increase in intrinsic apoptosis pathway activation in endothelial cells

**Fig. 6:**
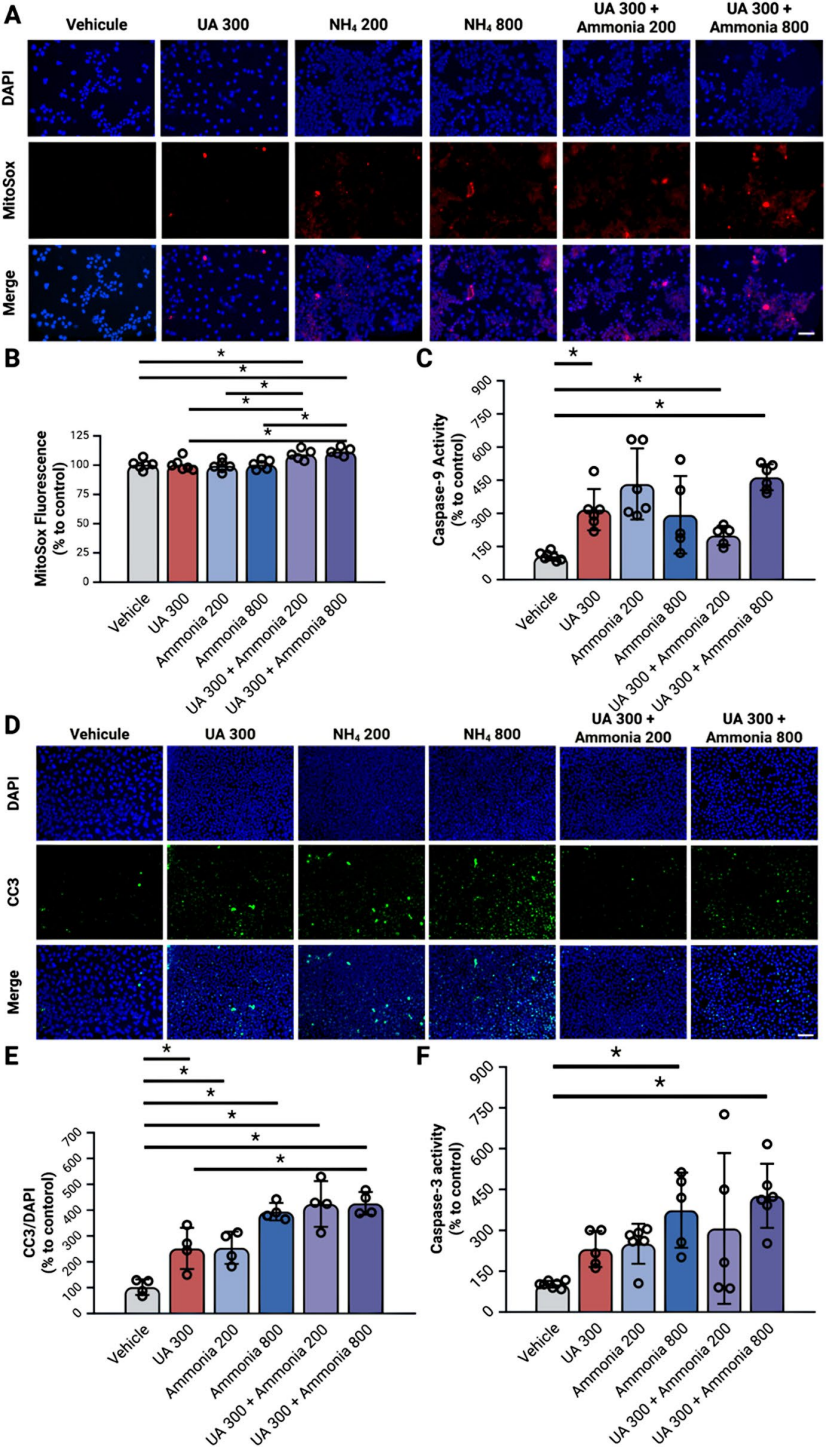
Combined hyperuricemia and hyperammonemia lead to the activation of the intrinsic apoptosis pathway. (**A**) MitoSox staining in rat brain microvascular endothelial cell cultivated on a microscope slide with a 12 well FlexiPerm insert and exposed to uric acid and ammonia for 20 h. (**B**) Quantification of MitoSox fluorescence measured on RBMEC cells cultivated in a 96 well plate and exposed to uric acid and ammonia for 20 h. (**C**) Enzymatic activity of caspase-9 in rat brain microvascular endothelial cells on cell lysates from a 6 well plate and exposed to uric acid and ammonia for 20 h. (**D**) Immunocytochemistry of cleaved caspase-3 in monolayer of culture rat brain microvascular endothelial cells on cell lysates from a 6 well plate and exposed to uric acid and ammonia for 20 h. (**E**) Quantification of cleaved-caspase-3 immunohistochemistry on a microscope slide with a 12 well FlexiPerm Insert and exposed to uric acid and ammonia for 20 h. (**F**) Enzymatic activity of caspase-3 in rat brain microvascular endothelial cells on cell lysates from a 6 well plate and exposed to uric acid and ammonia for 20 h. *N* = 4–6 per condition done in 2 replicates *p < 0.05 One-Way ANOVA with Tukey’s or Games-Howell post-hoc; Scale = 50 um; C3: caspase-3, CC3: cleaved caspase-3, C9: caspase-9, NH_4_: ammonia, RBMEC: rat brain microvascular endothelial cells, UA: uric acid. Created with BioRender

To clarify which endothelial cell loss pathways are implicated in the decreased viability associated with UA and ammonia exposure we then investigated the activation of the endothelial apoptosis pathway. The combination of UA 300 with ammonia 200 or 800 leads to a significant increase in MitoSox staining compared to vehicle, UA 300 alone or ammonia (200 or 800) alone (Fig. 6A-B). The combination of UA 300 with ammonia 200 or 800 leads to a significant increase in caspase-9 activity compared to vehicle (Fig. 6C).

All treatment conditions when compared to vehicle show a significant increase in cleaved-caspase-3 immunostaining (Fig. 6D-E). Ammonia 800 alone and UA 300 + ammonia 800 show a significant increase in caspase-3 activity when compared to vehicle (Fig. 6F).

### Loss of endothelial layer integrity in response to uric acid and ammonia in the context of hepatic encephalopathy

As a final marker of BBB integrity loss, we then investigated the expression of ZO-1 as a marker for tight junction integrity loss both in BDL rats and in RBMECs exposed to both UA and ammonia.

#### Significantly decreased cortical expression of ZO-1 is detected in bile-duct ligated rats with hyperuricemia

**Fig. 7:**
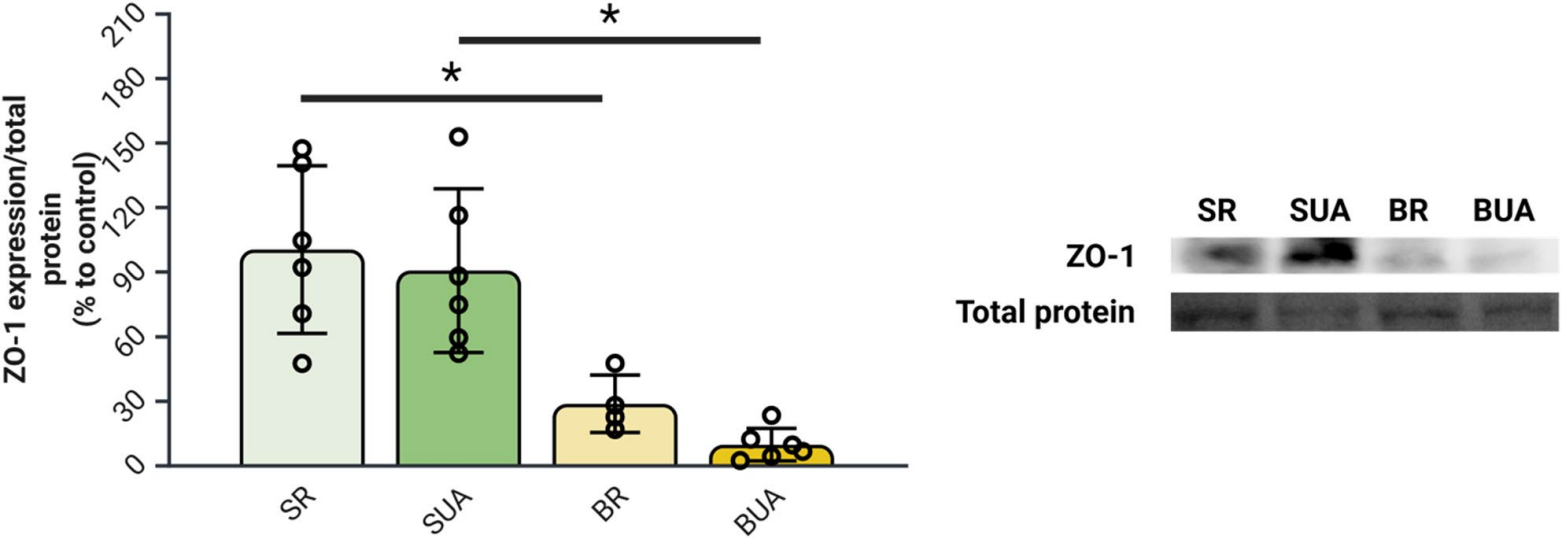
Hyperuricemia in bile-duct ligated rats leads to decreased expression of cortical zonula occludens 1. Evaluation by Western Blot of cortical ZO-1 expression in rats. *N* = 5–6 per group; *p < 0.05 Two-way ANOVA; BR: BDL + regular diet, BUA: BDL + high uric acid diet, SR: SHAM + regular diet; SUA: SHAM + high uric acid diet; UA: uric acid, ZO-1: zonula occludens 1. Created with BioRender

The BDL model leads to a significant decrease in cortical ZO-1 expression in both BR and BUA animal groups when compared to their counterparts (Fig. 7). However, the high-uric acid diet doesn’t lead to further decrease in ZO-1 expression BUA rats compared to BR rats (Fig. 7).

#### Co-exposure to uric acid and ammonia leads to a significant decrease in ZO-1 expression in endothelial cells

**Fig. 8:**
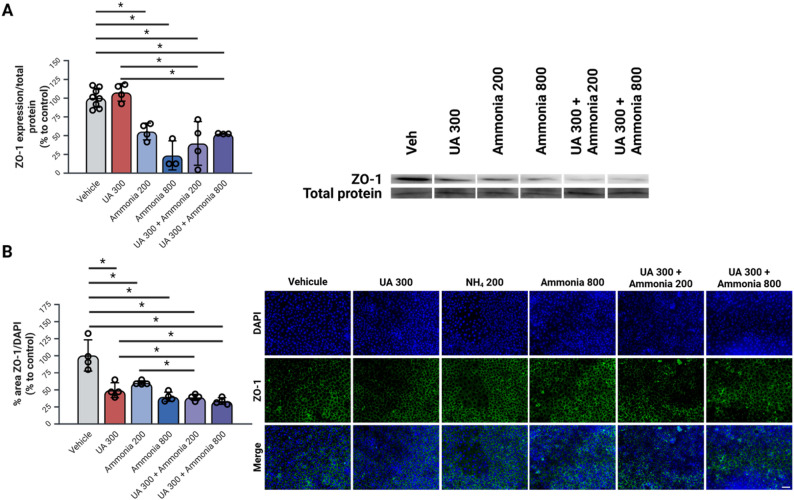
Combined uric acid and ammonia leads to a decreased expression of zonula occludens 1 in rat brain microvascular endothelial cells. (**A**) Western Blot analysis of ZO-1 expression in RBMEC cells cultivated in a 6 well plate and exposed to uric acid and ammonia for 20 h. (**B**) Immunocytochemistry of ZO-1 on a monolayer of RBMEC cells cultivated on a microscope slide with 12 well FlexiPerm inserts and exposed to uric acid and ammonia for 20 h. *N* = 4 per condition done in 2 replicates; *p < 0.05 One-Way ANOVA with Tukey’s post-hoc; Scale = 50 um; NH_4_: ammonia, RBMEC: rat brain microvascular endothelial cells, UA: uric acid, ZO-1: Zonula Occludens 1. Created with BioRender

All treatment conditions show a significant decrease in ZO-1 expression both for Western Blot analysis and for immunohistochemistry (Fig. 8A-B). Additionally, the combined treatments of UA and ammonia lead to a significant decrease in ZO-1 expression compared to UA 300 mmol/l alone for both Western Blot analysis and immunohistochemistry (Fig. 8A-B).

## Discussion

This study demonstrates that the combination of uric acid (UA) and ammonia, either in the pathological context of hepatic encephalopathy (HE) in bile duct-ligated (BDL) rats or applied directly to rat brain microvascular endothelial cells (RBMECs), leads to significant impairments of blood-brain barrier (BBB) integrity.

In BDL rats the administration of a high UA diet (BUA) leads to elevated cortical UA concentrations, correlated with plasma UA levels. This correlation suggests passive diffusion of UA between the blood and brain, implying a breakdown of BBB integrity. In healthy individuals, UA cannot cross the BBB [24], and in situ synthesis of UA in the brain is minimal due to the low expression of xanthine oxidase by cerebral cells [25]. These findings validate the use of cortical UA concentrations as a marker of endothelial layer disruption in BUA rats. Although well-validated dyes such as sodium fluorescein (NaF) or Evans’ Blue are commonly used to assess BBB permeability [26], their application in BDL models has produced inconsistent results. Some studies report BBB alterations [7, 26], while others do not [27]. For this reason, dye-based assays were not employed in the present study.

In RBMECs, the combination of UA and ammonia (200 and 800 µmol/L) lead to a significant decrease in TEER and an increase in NaF permeability. In the context of an ischemia-reperfusion injury, hyperuricemia lead to a significant decrease in intestinal TEER [28], but no evidence of hyperuricemia affecting BBB TEER was ever described in the litterature. Additionally, as seen in the present study, ammonia alone did not affect BBB resistance, highlighting the combined effects of UA and ammonia as inducing significant injury on the endothelial layer of the BBB.

Furthermore, the plasma concentrations of NO are significantly decreased in both SUA and BUA rats, confirming the role of UA in inducing endothelial dysfunction [17] even when looking at cerebral microvessels. UA is known for scavenging NO and decreasing endothelial NO synthetase activity leading to a decreased NO production [17]. Since NO is essential for communication and maintenance of the endothelial barrier, a loss of NO through scavenging by UA could contribute to the decreased endothelial cell viability that we observed.

The implication of UA in decreasing the bioavailability of NO is confirmed by the measure of NO in the cell culture media of UA treated RBMEC cells. Additionally, high concentrations (800 µmol/L) of ammonia alone lead to a significant decrease in NO concentration in the cell culture media of RBMEC cells. This goes against what is normally described in the literature with hyperammonemia associated with an increased NO synthesis [29].

To validate the underlying mechanisms associated with the BBB breakdown in both BUA rats and RBMEC cells treated with UA and ammonia, we then investigated 2 main factors that may impact BBB integrity: endothelial cell viability/loss and the integrity of tight junction proteins, focusing on ZO-1 due to the known effects of UA on this protein [28].

In BUA rats, the expression of CD31/PECAM-1, a specific marker of endothelial cells, was significantly decreased. PECAM-1 mediates cell to cell interactions and its downregulation is associated with endothelial cell dysfunction or damage [30]. Additionally, a significant co-immunostaining of two endothelial cell markers (CD31/PECAM-1 or Erg) and CC3 showed a significant increase in endothelial cells apoptosis in BUA rats. The apoptosis pathway involved in this endothelial cell loss was not further investigated in the cortex of BUA rats, but both UA and ammonia can induce mitochondrial damage [31, 32] activating the intrinsic apoptosis pathway [33]. This could be confirmed by co-immunostaining between endothelial markers and cleaved-caspase-9 in the cerebral cortex of BUA rats [34].

In RBMEC cells exposed to combined treatments of UA and ammonia, a significant decrease in cell viability is observed. This evident cell loss is mainly precipitated by the activation of the intrinsic apoptosis pathway triggered by the increase in mitochondrial oxidative stress [35], as measured by the MitoSox staining in this study. Ammonia alone is known to induce a pro-oxidant environment which could then initiate the conversion of UA from an antioxidant to a pro-oxidant molecule [21], explaining why UA alone does not increase mitochondrial oxidative stress. The following step of the intrinsic apoptosis pathway is the release of the cytochrome c from the mitochondria which is easily measured using a variant of the Western Blot technique [36]. However, this step was not assessed in RBMEC cells exposed to UA and ammonia. The following step of the intrinsic apoptosis pathway, the activation of caspase-9 [35], was then measured. Our data shows an increased caspase-9 activity in both groups treated with combination treatments of UA and ammonia. This further supports the conclusion that the activation of the intrinsic apoptosis pathway leads to endothelial cell loss in cells exposed to UA and ammonia. Finally, both the activity and immunostaining of cleaved-caspase-3 are increased in RBMECs exposed to UA and ammonia confirming the cells will undergo apoptosis. Indeed, caspase-3 is one of the effector caspases whose activation leads directly to apoptosis [35].

It is interesting that high ammonia (800 µmol/L) concentrations, are associated with a decreased endothelial cell viability and increased caspase-3 activity. However, this caspase-3 activation is not associated the intrinsic apoptosis pathway. This could be explained by the fact that high concentrations of ammonia alone induce an inflammatory response [8] and this inflammation could trigger the extrinsic apoptosis pathway [35]. To confirm this hypothesis, the expression of inflammatory cytokines in the culture media and the activity of caspase-8 could be evaluated.

In addition to the increased endothelial cell loss by apoptosis in BUA animals, an effect of the BDL surgery was also detected in both BR and BUA rats leading to a decreased expression of ZO-1. Based on these results, it could be concluded that where UA seems to have a more defined effect on endothelial cell viability, ammonia is more implicated in the alterations of tight junction complexes more precisely the expression of ZO-1. Indeed, in the intestinal epithelium, hyperammonemia was associated with a significant decrease in tight junction protein expression [31].

To validate these results, the expression of ZO-1 was measured by Western Blot and immunohistochemistry in cells exposed to ammonia alone and to combined treatments of UA and ammonia. Both concentrations of ammonia (200 and 800 µmol/L) as well as combined treatments of UA and ammonia lead to a significant decrease in ZO-1 expression. These results further support the implication of hyperammonemia in paracellular permeability increase in the endothelial layer of the BBB which is also a validated conclusion in the literature [37].

We acknowledge that our study present limitations in its design and findings, that might influence our conclusions. Particularly, the use of a monolayer RBMEC instead of a more complex model using co-culture with astrocytes and pericytes, known to modulate and stabilize the BBB [1, 38], poses a limitation to the chosen BBB model. Additionally, the discrepancy between the permeability findings and the combined effects of UA and ammonia on viability might be driven by different seeding conditions affecting the polarization of the endothelial layer. However, these two parameters still align in their conclusion, even though the reported permeability changes may not fully capture the cellular injury processes. A final limitation of our study is its descriptive nature explaining the mostly causal observations. Further studies focusing on rescue capabilities of the BBB after a UA and ammonia insult would bring deeper understanding into the potential synergistic effects of these two molecules on the BBB.

In conclusion, the combination of UA and ammonia both in BDL rats and in a monolayer RBMEC culture model leads to significant impairments of paracellular BBB integrity. Exposure to uric acid leads to a significant decrease in bioavailability of NO contributing to an increase in endothelial cell loss through the activation of the intrinsic apoptosis pathway. On the other hand, ammonia causes a dysfunction of tight junction protein complexes, by affecting the expression of ZO-1, leading to an increase in paracellular permeability. These results highlight the importance of further studies into the combined effects of UA and ammonia in unlocking the BBB of cirrhosis patients potentially contributing to irreversible cerebral lesions.

## Data Availability

The data that support the findings of this study are available from the corresponding author upon reasonable request.

## Abbreviations

BBB: Blood-brain barrier
BDL: Bile-duct ligation
BR: BDL-regular diet
BUA: BDL-high uric acid diet
C3: Caspase-3
CD31/PECAM-1: Platelet and endothelial cell adhesion molecule 1
Erg: Vascular endothelial transcription factor
NaF: Fluorescein sodium salt
NH_4_: Ammonia
NO: Nitric oxide
RBMEC: Rat brain microvascular endothelial cells
SHAM: Control
SR: SHAM-regular diet
SUA: SHAM-high uric acid diet
TEER: Transendothelial resistance
UA: Uric acid
ZO-1: Zonula occludens-1
